# Supplementary material for: A mitochondrial DNA hypomorph of cytochrome oxidase specifically impairs male fertility in Drosophila melanogaster
Source: eLife. 2016 Aug 2;5:e16923. doi: 10.7554/eLife.16923 (PMC4970871; doi:10.7554/eLife.16923)
Supplement: Figure 4—source data 1. — Summary of data from duplex sequencing of COIIG177S mutant and wildtype mtDNA strains re-isolated from the ancestral, heteroplasmic w1118 stock. Individual fly heads from the mutant COIIG177S mtDNA stock (Mut Fly 1, 2, 6–10) were subjected to duplex sequencing. Pooled samples represent sequencing data generated by sequencing combined DNA from 10 individuals from either the mutant or wildtype mtDNA strains. DOI: http://dx.doi.org/10.7554/eLife.16923.011 [file elife-16923-fig4-data1.docx]

| **Sample ID** | **Depth @ Position 3611** | **# G177S Variant**  **Reads** | **# Wild-type Reads** | **% Variant** |
| --- | --- | --- | --- | --- |
| Mut Fly 1 | 11,230 | 11,230 | 0 | 100 |
| Mut Fly 2 | 8,533 | 8,533 | 0 | 100 |
| Mut Fly 6 | 9,329 | 9,329 | 0 | 100 |
| Mut Fly 7 | 10,484 | 10,484 | 0 | 100 |
| Mut Fly 8 | 11,076 | 11,069 | 7 | 99.9 |
| Mut Fly 9 | 12,103 | 12,103 | 0 | 100 |
| Mut Fly 10 | 10,303 | 10,303 | 0 | 100 |
| Mut Fly Pool | 7,061 | 7,061 | 0 | 100 |
| WT Fly Pool | 7,209 | 0 | 7,209 | 0 |

**Patel et al.,**

**Figure 4 supplement 1**
